# Mechanism and Application of Chitosan and Its Derivatives in Promoting Permeation in Transdermal Drug Delivery Systems: A Review

**DOI:** 10.3390/ph15040459

**Published:** 2022-04-10

**Authors:** Jinqian Ma, Yuchen Wang, Rong Lu

**Affiliations:** Marine College, Shandong University, Weihai 264209, China; 17854160138@163.com (J.M.); w17660520116@163.com (Y.W.)

**Keywords:** chitosan and its derivatives, transdermal administration, permeation promotion, mechanism, application

## Abstract

The mechanisms and applications of chitosan and its derivatives in transdermal drug delivery to promote drug permeation were reviewed in this paper. Specifically, we summarized the permeation-promoting mechanisms of chitosan and several of its derivatives, including changing the structure of stratum corneum proteins, acting on the tight junction of granular layers, affecting intercellular lipids, and increasing the water content of stratum corneum. These mechanisms are the reason why chitosan and its derivatives can increase the transdermal permeation of drugs. In addition, various transdermal preparations containing chitosan and its derivatives were summarized, and their respective advantages were expounded, including nanoparticles, emulsions, transdermal microneedles, nanocapsules, transdermal patches, transdermal membranes, hydrogels, liposomes, and nano-stents. The purpose of this review is to provide a theoretical basis for the further and wider application of chitosan in transdermal drug delivery systems. In the future, research results of chitosan and its derivatives in transdermal drug delivery need more support from in vivo experiments, as well as good correlation between in vitro and in vivo experiments. In conclusion, the excellent permeability-promoting property, good biocompatibility, and biodegradability of chitosan and its derivatives make them ideal materials for local transdermal drug delivery.

## 1. Introduction

The skin is composed of three parts, namely, the epidermis, dermis, and subcutaneous tissues from outside to inside. The epidermis is divided into the stratum corneum, transparent layer, granular layer, spinous layer, and basal layer from outside to inside [1,2,3]. The stratum corneum is located on the shallowest surface of the skin and is composed of multiple layers of dead keratinocytes. It has the effects of moisturizing, anti-friction, preventing the extravasation of tissue fluid from the body, and preventing the invasion of chemicals and bacteria into the body [4,5]. Designing the most appropriate delivery route for pharmaceutical preparations has long been an important research topic in the field of drug delivery. As the organ with the largest mass and surface area of the human body, the skin is undoubtedly the ideal delivery route for many drugs [6,7,8]. Compared with other administration routes such as intravenous administration and oral administration, skin administration has the advantage of avoiding the first-pass effect, long administration interval, stable blood concentration, realizing local or systemic administration, etc. [9,10], However, skin disorders represented by the stratum corneum largely limit the percutaneous absorption of many drugs. Therefore, how to achieve efficient transdermal drug delivery to achieve better therapeutic effects is the main challenge facing researchers.

The existing common transdermal preparations mainly comprise patches, ointments, spreads, sprays, and the like [11,12,13,14], These preparations are widely used for the treatment of skin disease. Laser devices in fractional mode have also been proposed in order to enhance the transdermal delivery of different drugs [15]. However, they have some disadvantages, including low transdermal penetration of drugs, low bioavailability, adverse reactions such as local stimulation of the skin, and poor compliance of patients [16,17,18,19,20]. Researchers are looking for various new auxiliary materials to address the above problems. Some synthetic high-molecular-weight polymers such as PEG and PLA have been used for transdermal drug delivery due to their excellent stability and adhesion properties [21,22,23,24,25]. However, the degradation inertness, cytotoxicity, and resulting immunogenicity of these synthetic high-molecular-weight polymers are problems that we must consider; accordingly, researchers tend to use high-molecular-weight polymers of natural origin such as polysaccharides. Polysaccharides are formed by condensation and dehydration of multiple monosaccharide molecules. They are carbohydrate substances with complex molecular structure and large size. Chitosan is a product of partially removing the acetyl of natural polysaccharide chitin, and it has multiple physiological functions such as inhibiting bacteria, resisting cancer, reducing blood fat, and enhancing immunity [26,27,28,29,30,31]. Chitosan and its derivatives have become a hot topic in research on transdermal drug delivery due to their good biocompatibility, biodegradability, and nontoxicity.

Chitosan is the product of *N*-deacetylation of chitin. Due to the simultaneous presence of amino, acetamido, and hydroxyl groups in the molecule, chitosan is quite active in nature and can be modified, activated, and coupled, showing rich functionality and modifiability in biological applications. As the only polycationic polymer of natural origin, chitosan is capable of interacting with negatively charged cell membranes to assist the loading of drugs across the cell membrane [32,33]. The polycationic nature of chitosan and its derivatives allows them to easily interact with the negative charge on the protein to facilitate protein drug encapsulation [34]. Chitosan and its derivatives can also chelate heavy-metal ions and are high-performance metal ion-trapping agents [35].

**Figure 1:**
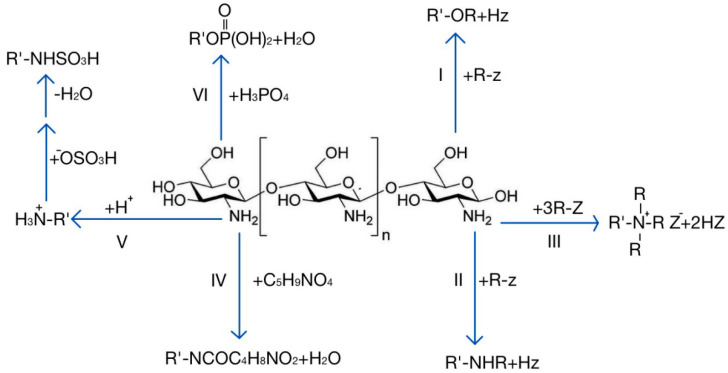
Several main modification reactions of chitosan. I: *O*-alkylation; II: *N*-alkylation; III: quaternization; IV: reaction with glutamic acid; V: sulfonation; VI: phosphorylation.

As mentioned above, chitosan is rich in active groups, which is conducive to achieving a variety of reactions to obtain chitosan derivatives with different characteristics. Carboxymethyl chitosan can be prepared through *N*-alkylation or *O*-alkylation, which shows improved water solubility and bacteriostasis and has good film-forming properties [36,37]. The *N*-acyl compound generated by the reaction of chitosan and glutamic acid has good moisture retention and adsorbability, and it can be used for absorbing metal ions [38]. The *N*-quaternary ammonium salt can be used for preparing polycationic polymers and enhancing the adhesion and adsorption capacity [39,40]. The product obtained by phosphorylation of chitosan can promote bone tissue regeneration and calcium absorption [41,42]. The chitosan sulfonation product has a heparinoid effect, has good bacteriostatic activity, and improves immunity [43,44,45]. These chitosan derivatives with different properties have been used for drug delivery research and have shown unique advantages. Figure 1 shows several major modification reactions of chitosan and the obtained products.

Chitosan and its derivatives can be degraded by physical methods [46,47,48], chemical methods [49,50], and enzymatic methods [51,52,53]. Chitosan involved in transdermal drug delivery is degraded in vivo by lysozymes, without the need of other reagents, and there is no generation of toxic byproducts in the reaction, thus representing the most ideal method of chitosan degradation. Lysozymes hydrolyze chitosan into low-molecular-weight, nontoxic chitosan oligosaccharides by acting on the β-1-4 glycosidic bond of the chitosan chain, and this degradation is not substrate-specific [54]; however, the enzymatic degradation of chitosan is related to its degree of deacetylation. Excessive deacetylation can result in chitosan being difficult to hydrolyze by lysozymes [55,56].

The mechanism of action of chitosan and its derivatives in transdermal drug delivery systems has been extensively discussed, and their specific application forms are constantly being developed. This review details the potential mechanisms of chitosan in drug transdermal delivery systems, including the transdermal mechanism and sustained release mechanism, and summarizes the latest progress in chitosan-related transdermal preparations.

## 2. The Mechanism of Chitosan and Its Derivatives in Promoting Drug Penetration

**Figure 2:**
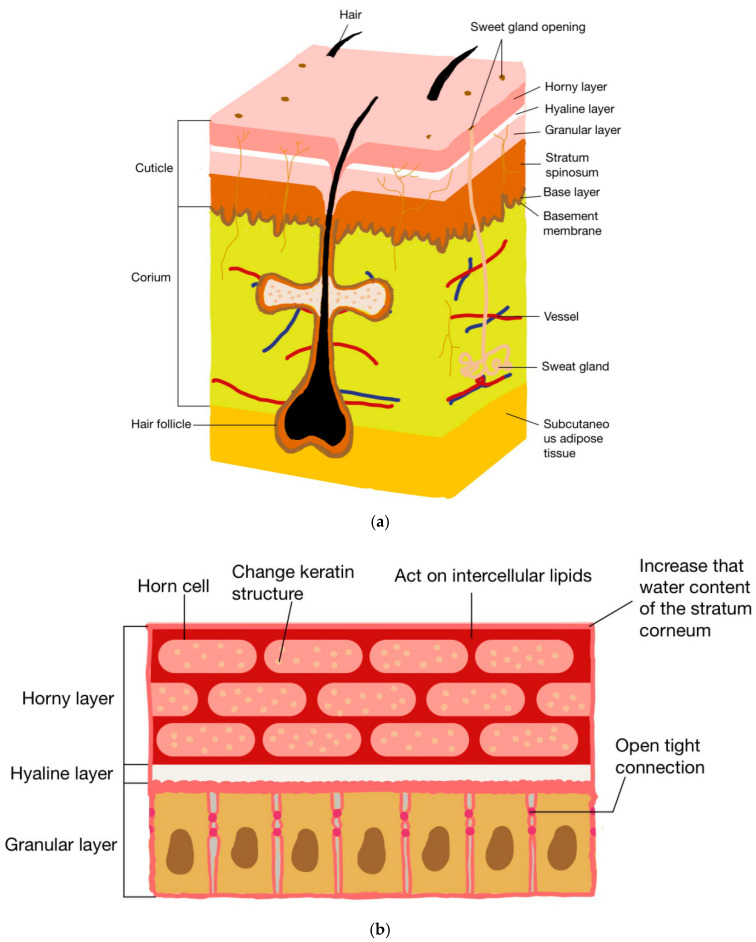
Skin structure and mechanisms of chitosan and its derivatives in promoting drug penetration: (**a**) structure of skin; (**b**) mechanism of chitosan and its derivatives in promoting drug penetration.

Drug transdermal penetration is affected by a variety of structures and components such as keratinocytes, keratin, intercellular lipids, and stratum corneum water content. Therefore, chitosan and its derivatives can promote drug penetration through a variety of mechanisms. Figure 2 shows the structure of the skin and the mechanisms via which chitosan and its derivatives promote drug penetration.

### 2.1. Changing the Structure of Stratum Corneum Protein

Keratins are structural proteins in keratinocytes and components of the cytoskeleton. A total of 20 cellular keratin proteins are distributed in various epithelial cells throughout the body. Together with keratinocytes and intercellular lipids, keratin forms the “brick-wall” structure of the stratum corneum, preventing foreign substances from entering systemic circulation through the skin. Therefore, changing the conformation of keratins can reduce the cohesion of the stratum corneum barrier and promote drug penetration. He et al. studied the permeation-enhancing mechanisms of *N*-trimethyl chitosan (TMC) and mono-*N*-carboxymethyl chitosan (MCC) with different quaternization degrees (DQ), and they detected the changes in the secondary structure of the proteins in the stratum corneum by attenuated total reflection Fourier-transform infrared spectroscopy (ATR-FTIR) [57]. The protein secondary structure of the stratum corneum can be reflected by the positions of the amide I absorption peak and the amide II absorption peak. It was found that, after treatment with CS, TMC, and MCC, the amide II absorption peak shifted to a lower wavelength relative to the amide I absorption peak, indicating that the protein secondary structure changed. According to previous research results, it can be speculated that the transdermal effect of CS and its derivatives is related to the changes in the secondary structure of proteins. In this study, the mechanism of transdermal enhancement of chitosan was demonstrated for the first time, which laid a foundation for in-depth studies on the penetration-enhancing effect of chitosan and its derivatives. Asif Nawaz et al. found lower wavenumbers of the FTIR peaks at 3355.80 cm^−1^, 2918.50 cm^−1^, 2850.10 cm^−1^, and 1548.50 cm^−1^ in CSN-treated skin compared to untreated skin, suggesting that the N–H/C–N forms of amide II in CSN-treated keratin were fluidized [58].

### 2.2. Tight Junction Acting in Granular Layer

The tight junction, also known as atretic zona, is a virtually impenetrable barrier of adjacent cell membranes. It is the area where two cells are closely connected. Tight junctions can prevent molecules and ions from passing through the intercellular space, further limiting the percutaneous absorption of drugs on the basis of the stratum corneum barrier.

Tzyy-Harn Yeh et al. explored the effect of chitosan on the tight junction of intestinal epithelial cells at the molecular level [59]. The results showed that, after treatment with chitosan, transmembrane proteins known as Claudins (CLDNs) were redistributed, and the tight junction between monolayers of Caco2 cells was opened instantaneously, as directly observed by confocal laser scanning microscopy (CLSM) and transmission electron microscopy (TEM). Western blot quantitative analysis revealed that, compared with the control group, CLDNs in the chitosan treatment group exhibited a time-dependent deletion, i.e., CLDNs were redistributed after chitosan was removed for a period of time. This study clarified the mechanism of chitosan in opening the tight junction of epithelial cells, i.e., by regulating the redistribution of CLDNs, whereby the tight junction could be instantaneously and reversibly opened, thus affecting epithelial permeability. It should be noted that, although the cells in this study were intestinal epithelial cells, in view of the fact that the tight junction of epidermal cells also contain CLDNs, chitosan and its derivatives might open the tight junction via the same mechanism. In addition, positively charged chitosan and its derivatives interact with negatively charged cell membranes to weaken the tightly coupled barrier action [60].

### 2.3. Interaction with Intercellular Lipids

Intercellular structural lipids are important components of the skin, including cholesterol, ceramide, and free fatty acid. Intercellular lipids can regulate the permeability of the stratum corneum barrier. When the skin is stimulated or suffers from metabolic disorders, the proportion of the above three main components of intercellular lipids changes significantly. On this basis, changing the ratio of the three lipid components can disturb the ordering of the stratum corneum, thereby increasing permeability. The study by Xueqin Zhou et al. found that low-molecular-weight chitosan could increase the transdermal administration of baicalin to mice [61]. Specifically, at pH 7.5, some amino groups in low-molecular-weight chitosan CS80-1000 were protonated, and their interaction with carboxylic acid groups in the stratum corneum proteins was weak, which could not be detected by ^13^C cross-polarization/magic angle spin nuclear magnetic resonance (^13^C-CP/MAS-NMR) spectroscopy. However, at pH 7.5, the transdermal effect of baicalin was significantly enhanced. Since the stratum corneum is mainly composed of protein and lipids, and since protein was not significantly affected, the permeability of baicalin enhanced by low-molecular-weight chitosan was speculatively attributed to its interaction with lipids. On this basis, they found that the enhancement degree of baicalin permeability was highly dependent on the concentration of CS80-1000, whereby CS80-1000 at a higher concentration showed a more significant enhancement degree of baicalin permeability.

### 2.4. Increasing the Stratum Corneum Water Content

The stratum corneum has the function of preventing water loss and storing water in the body to maintain the physiological function of the skin. The moisture content of the stratum corneum affects skin elasticity, enzyme activity in the stratum corneum, skin roughness, and transdermal permeability of the drug. A higher moisture content of the stratum corneum results in better transdermal permeability of the drug. Chitosan is a rigid structure that limits the passage of a large number of water molecules. However, modified chitosan derivatives can change this property. Satheeshabu et al. attached thioglycolic acid to chitosan via an acylation reaction between a primary amino group and a carboxylic acid group to form conjugated chitosan, and the change in the conversion of the primary amino group of chitosan to a thiol group allowed the opening of the polymeric chain to form a loose matrix, allowing more water molecules to pass through [62]. The results of the moisture absorption study showed that the moisture absorption of the patch increased with the increase in the proportion of conjugated chitosan in the matrix. The in vitro permeability test also validated the result that the drug permeability of the patch containing conjugated chitosan was higher than that of the patch containing only chitosan. He et al. detected the water content of the stratum corneum using instrumental analysis [57]. In ATR Fourier-transform infrared spectroscopy, the peak of amide I represents the absorbance of water and protein in the skin, while that of amide II represents the absorbance of protein in the skin. Therefore, the ratio of amide I and amide II peaks can be used to indicate the proportion of water in the skin. They treated the isolated mouse skin with solutions of chitosan with different quaternization degrees and mono-*N*-carboxymethyl chitosan, and they found that, compared with the blank solvent, all chitosan derivatives significantly increased the water content of the stratum corneum within 8 h, i.e., the ratio of amide I and amide II peaks was significantly increased before gradually decreasing. After water entered the stratum corneum from the solution, the three-dimensional network structure of chitosan and its derivatives could serve as a storage space for water in the stratum corneum for a long time.

## 3. Application of Chitosan and Derivatives in Transdermal Drug Delivery Systems

**Figure 3:**
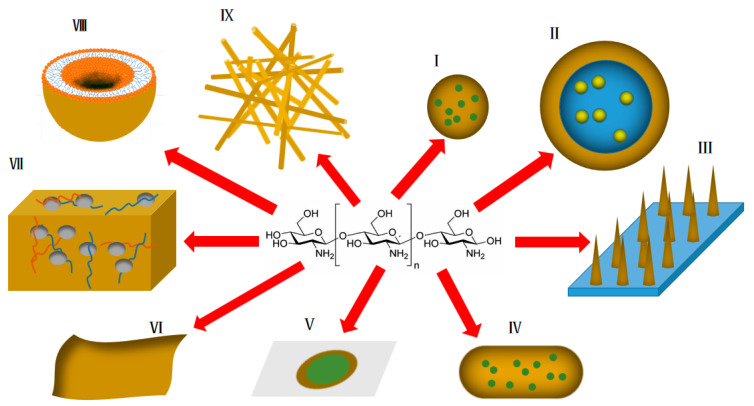
Various forms of application of chitosan and its derivatives in transdermal drug delivery systems (yellow represents chitosan and derivatives thereof). I: nanoparticles; II: emulsions; III: transdermal microneedles; IV: nanocapsules; V: transdermal patches; VI: transdermal membranes; VII: hydrogels; VIII: liposomes; IX: nano-scaffolds.

As mentioned above, chitosan and its derivatives have unique advantages in transdermal drug delivery systems. Some practical application forms of chitosan and its derivatives in transdermal drug delivery research (Figure 3) are summarized below.

### 3.1. Nanoparticles

Nanoparticle refer to artificial solid particles with at least one dimension in the nanometer size range (0.1–100 nm) in three-dimensional space, also known as nano-dust or nano-powder. The ultrasmall size of nanoparticles enables them to have extraordinary permeability and great specific surface area, thus having great application value in transdermal drug delivery.

As the matrix of drug-loaded nanoparticles, chitosan has the advantages of sustained and controlled release, adhesion, permeability, and biocompatibility. Alaa Riezk et al. prepared amphotericin B-loaded chitosan nanoparticles for the treatment of cutaneous leishmaniasis [63]. Two amphotericin B chitosan nanoparticles with different electrical properties were prepared using positively charged sodium tripolyphosphate (TPP) and negatively charged dextran sulfate as crosslinkers. These two nanoparticles both showed high in vitro activity against *Leishmania* amastigotes. In addition, amphotericin B in aqueous solution could not penetrate the skin. When two types of AmB-loaded chitosan nanoparticles were applied to isolated mouse skin, AmB could slowly and limitedly penetrate the skin, and osmotic balance was achieved after about 20 h, indicating that chitosan nanoparticles could promote the in vitro skin permeation of amphotericin B. Delaying drug release is another effect of chitosan matrices containing nanoparticles. Salma et al. prepared tacrolimus-loaded chitosan nanoparticles using ion gelation technology for the treatment of psoriasis [64]. The in vitro skin permeation was determined over 24 h. The permeability of tacrolimus in the chitosan nanoparticles after 24 h was 24%; correspondingly, the permeability of tacrolimus cream after 24 h was 61%, i.e., the chitosan nanoparticles could significantly delay the release of tacrolimus and reduce the systemic toxicity of the drug. In addition, the 24 h skin deposition rate of tacrolimus cream was 11.4%, while that of the tacrolimus chitosan nanoparticles reached 75%, representing a benefit for the delivery of tacrolimus to the skin, which is the target site for the treatment of psoriasis. Psoriasis leads to skin sclerosis at the lesion site, greatly impeding the transdermal delivery of drugs. In this study, chitosan nanoparticles could improve the transdermal permeability of tacrolimus, delay its release from the cortex, prolong its action time at target sites, and reduce the effect of drugs on the whole body. Rada Al-Kassas et al. prepared a transdermal propranolol delivery system [65] with chitosan nanoparticles dispersed in gel, and they used porcine ear skin similar to human skin for the permeation study. The results showed that the system had thixotropy and slow-release effects, which were related to the local formation of a drug reservoir after chitosan nanoparticles were absorbed by skin. However, this conclusion requires further in vivo research support. Chitosan nanoparticles can neither increase nor reduce the toxicity of drugs toward skin keratinocytes and fibroblasts, which is a safe transdermal drug delivery system [66].

### 3.2. Emulsions

Emulsions are two-phase liquids that are immiscible with each other. One phase is dispersed as small droplets in the other phase to form a nonuniformly dispersed liquid formulation. Insoluble medicine is prepared into emulsions with large liquid drop dispersion, fast medicine absorption and drug effect exertion, and high bioavailability. Topical emulsions can improve permeability of the skin and mucosa, as well as reduce irritation. When in use, emulsions are uniformly coated on the medicinal part.

Chitosan mainly acts as a coating in transdermal milk. As a cationic polymer, chitosan and its derivatives can be coated on the outer layer of the emulsion to interact with the anionic surfactant in the emulsion through electrostatic interaction, thereby protecting the emulsion from agglomeration and maintaining its stability [67]. Taif Ali Khan et al. prepared a chitosan-coated 5-fluorouracil transdermal emulsion which showed good skin permeation characteristics compared with the 5- fluorouracil solution, which was related to the fluidization of the stratum corneum by chitosan and the surfactants in the emulsion, as described previously. Zeta potential is an important parameter representing the stability of an emulsion. A higher absolute value of the zeta potential indicates a more stable system. Zeta potential results showed that the chitosan-coated emulsion system had a higher positive zeta potential (+3.9 mV), i.e., the chitosan coating enhanced the stability of the emulsion system [58].

The mucosal adhesion of chitosan can prolong the retention time of an emulsion and facilitate continuous penetration. Babita Kumari et al. studied the effect of chitosan coating on the skin permeation properties of clotrimazole microemulsion [68]. The drug retention in rat skin after 8 h transdermal permeation was measured, and the results showed that the chitosan-coated clotrimazole microemulsion had higher drug retention in the skin compared with the control clotrimazole microemulsion (*p* < 0.05). Asma Sharkawy et al. used a chitosan/acacia clindamycin emulsion as the carrier to deliver *trans*-resveratrol, and the skin penetration test results showed that resveratrol had a high retention level in epidermis and dermis, which is of great significance for reducing the dosing time [69].

### 3.3. Transdermal Microneedles

Transdermal microneedles are microneedles that open the skin pipeline using micro- or nanotechnology, with the dual characteristics of injection administration and transdermal administration. When in use, as long as a small chip covered with microneedles is pasted on the skin, the microneedles can penetrate the cuticle layer of the epidermis, which functions as a barrier for drugs. A patch filled with drugs pasted on the skin allows their slow penetration into the epidermis, allowing them to be quantitatively and continuously administered. Compared with other transdermal preparations such as transdermal patches, transdermal membranes, creams, and the like, transdermal microneedles can directly penetrate the stratum corneum barrier to be administered to the dermis layer, have little extracutaneous residue, and can almost fully cross the stratum corneum, thereby greatly improving the skin permeation and absorption rate of the medicine [19,70]. Microneedles entering the skin can serve as a local drug reservoir to achieve continuous drug administration. Meanwhile, compared with traditional injection, transdermal microneedles avoid the pain caused by puncturing the skin when using a needle [71], which improves the tolerance of patients. In addition, since a large skin wound does not form, the risk of infection by injection is greatly reduced [72].

Water-soluble microneedles such as water-soluble polyvinylpyrrolidone (PVP), polyvinyl alcohol, and carboxymethyl cellulose (CMC) are rapidly dissolved after entering the skin, resulting in excessive drug release, while chitosan and its derivatives with relatively low water solubility can avoid this problem [34]. In addition, chitosan and its derivatives have good biocompatibility and biodegradability. Microneedles made of these materials do not need to be taken out after drug administration and can be degraded harmlessly in vivo. Zulcaif Ahmad et al. prepared a thiolated chitosan microneedle patch for transdermal delivery of tacrolimus, which achieved a high skin penetration efficiency (84%) and a longer release time (48 h 53.8 7.30%), with the sustained-release property related to the slow degradation of the needle after the microneedles entered the skin [73]. Mei-Chin Chen et al. developed a chitosan microneedle patch for the delivery of macromolecular drugs. The chitosan solid microneedles prepared by the double-casting method had sufficient mechanical strength to be inserted into pig skin at a depth of 250 μm in vitro and into rat skin at a depth of 200 μm in vivo. In vitro drug release studies showed the sustained release of BSA from chitosan microneedles with a cumulative release of approximately 95% observed over 8 days. This study confirmed that chitosan transdermal microneedles could achieve the skin delivery of macromolecules, which has significance for the development of transdermal delivery of biological macromolecules. On the basis of this study, the research group also investigated the possibility of chitosan microneedles as vaccine delivery vehicles. They developed a fully embedded chitosan transdermal microneedle. The delivery structure design enables the insertion array and the support array to be separated automatically after the microneedle enters the skin, thus realizing the sustained delivery of drugs for a long time without patches. Studies have shown that the microneedle system can prolong antigen exposure at the insertion site for up to 14 days and induce a robust immune response for at least 6 weeks [74].

Chitosan with natural anti-inflammatory and antibacterial properties can be used to promote skin wound healing. Junjie Chi et al. prepared a chitosan microneedle patch for promoting skin wound healing [75]. The wound models were treated with chitosan microneedle patches, microneedle-free patches, planar chitosan membranes, and PBS. The results showed that the expression levels of two proinflammatory factors (IL-6 and TNF-α) in the chitosan microneedle patch group were the lowest, indicating that, compared with other groups, the chitosan microneedle patch had the best effect on skin wound healing. Chitosan itself has a certain anti-inflammatory and antibacterial effect. The structural design of microneedles enhances the delivery efficiency of anti-inflammatory and antibacterial drugs, on one hand, and promotes the gas exchange between the outside world and regenerated tissues, as well as a reduction in the levels of inflammatory factors as compared with the planar chitosan membrane, on the other hand.

### 3.4. Nanocapsules

Drugs can be encapsulated in a capsule wall formed by inorganic or organic polymeric materials using microencapsulation technology to form a core–shell structure, which can isolate the drug active ingredient from the outside in the delivery process to avoid degradation and inactivation, with subsequent release upon reaching the target site. Microcapsules with a particle size of less than 1 μm are commonly referred to as nanocapsules. Nanocapsules not only have the effects of embedding, targeting, and sustained release, but can also improve the dispersibility, solubility, and stability of nanoparticles. In terms of transdermal drug delivery, nanocapsules can increase the permeability and adhesion of drugs to enhance the therapeutic effect.

María Javiera Alvarez-Figueroa et al. designed and prepared imiquimod-loaded chitosan nanocapsules for transdermal drug delivery, with an average particle size of 200 nm and a PDI less than 0.3, indicating a uniform system. The nanocapsules were stable under physiological conditions for at least 48 h, and the stability was good. The permeability of the nanocapsules was studied with pig skin. Raman spectrum analysis showed that the imiquimod band was observed at a depth greater than 20 μm, confirming that the nanocapsules could penetrate the stratum corneum and serve as an ideal platform for the transdermal delivery of imiquimod; furthermore, it was clear that chitosan played an important role in promoting permeability and isolating [76]. In addition, the mucoadhesive property of chitosan may also be responsible for the high permeability of the coated drug [77].

### 3.5. Transdermal Patches

Transdermal patches refer to thin-film preparations that can be pasted on the skin, where drugs enter the blood circulation through the skin. Transdermal patches consist of a backing layer, a drug reservoir, an adhesive layer, and a protective layer removed before use. The backing layer is located on the outermost side for isolating and protecting the drug delivery system from the outside world. The drug storage libraries are located in the middle. Some drug storage libraries also have controlled-release membranes to achieve sustained and controlled release of drugs. The adhesive layer is located on the innermost side and comes into direct contact with the skin for fixation. Compared with other transdermal preparations, the patch has the advantage that the drug can be stably retained on the skin for a long time without loss, ensuring the sustainability and stability of drug administration.

Chitosan and its derivatives are mostly used as dressing matrices in the dosage form of transdermal patches. Due to their good biocompatibility and biodegradability, chitosan transdermal patches are superior to other materials in promoting wound healing. The anti-inflammatory and antibacterial effects of chitosan can be synergistic with the delivered drugs. Specifically, Cs can accelerate the proliferation of fibroblasts and granulation, increase analgesic and hemostatic effects, stimulate neutrophils and IgM, interact with membrane phospholipid molecules, enhance the activation of macrophages and the production of extracellular matrix, and enhance antibacterial activity. Patches may also absorb excess inflammatory exudate to maintain a clean wound environment [78]. In addition, chitosan also has water retention property, which is of great significance for the water demand during the process of skin tissue repair and regeneration. Arash Ghalayani Esfahani et al. designed an electrophoretic deposition chitosan patch for local drug delivery. Using the water solvent, hydrogen was generated at the cathode electrochemical interface to form a porous structure. The use of the preparation to deliver the fat-soluble drug clobetasol propionate could achieve 80% drug release within 2 h, meeting the needs of local drug delivery [79]. In addition, chitosan could prolong the adhesion and release of the patch and reduce the drug administration time. Niranjan et al. prepared a PVA/curcumin/chitosan patch to improve the bioavailability of the water-insoluble drug curcumin [80]. The rat skin wound model was used to study the therapeutic performance of the patch. Compared to the corresponding commercially available creams, the PVA/curcumin/chitosan patch resulted in faster wound healing, manifested as uniform hair growth and reduced wound area in the dermal area. This was attributed to the fact that the sustained-release property of chitosan could reduce the number of patch replacements, which was conducive to accelerating wound healing.

### 3.6. Transdermal Membranes

Film agents refer to the raw materials of drugs and appropriate film-forming materials processed into film-like preparations. The film can be used for oral, sublingual, vaginal, and other mucosal administration, and they can also be used for covering skin wounds and inflammatory surfaces. The active ingredient can be uniformly dispersed by preparation of the medicine into a film agent, so that the medicine can be accurately and quantitatively administered, as well as quickly dissolved, which facilitates absorption. In addition, according to different requirements, different matrix materials can be adopted to prepare the film agent with different release rates, so that the controlled release of the drug is realized. As a natural polysaccharide, chitosan has good ductility, biodegradability, and multifunctional properties, making it an ideal material for transdermal membrane agents. Rishabha Malviya et al. prepared a chitosan-based polysaccharide complex transdermal membrane [81]. They evaluated a series of physicochemical properties of the transdermal membrane, including drug content, water content, and swelling property. The results showed that the average water content of the membrane was 3.08% to 2.44%, and the drug content was 95.81% ± 0.75% to 98.08% ± 0.88%. The swelling coefficient was decreased, and the solubility of chitosan was increased by the addition of an ionic solvent. However, they did not study skin permeation in vivo; therefore, the actual application effect of the chitosan transdermal membrane needs to be further tested and evaluated. The abundant functional groups in the structure of chitosan allow it to react with many molecules to produce modified bodies with various properties, some of which have properties that chitosan does not have, thus meeting the needs of different applications. Sonia Trombino et al. combined cyclosporin A with chitosan carboxylate through an amidation reaction to obtain a prodrug, which was uniformly dispersed in a chitosan-based polymer membrane for the treatment of breast cancer [82]. The membrane was analyzed by differential scanning calorimetry (DSC) and scanning electron microscopy (SEM). The results showed that an amide bond was effectively formed between cyclosporin A and chitosan carboxylate, and cyclosporin A was completely dispersed in the polymer membrane. SEM micrographs showed that the membrane structure formed by the chitosan matrix and chitosan carboxylate-cyclosporin A was compact. The results of skin permeation experiments conducted with porcine skin showed that the model drug coumarin-6 could penetrate into the dermis, going beyond the surface and deeper into the tibia. However, more studies are needed to verify the permeability of the membrane.

### 3.7. Hydrogels

Hydrogels are extremely hydrophilic gels with a three-dimensional network structure. They rapidly swell in water and can maintain a large volume of water without dissolution or deformation in the swollen state. Due to the existence of a crosslinked network, hydrogels can swell and retain a large amount of water. The water absorption is closely related to the degree of crosslinking. A higher degree of crosslinking results in lower water absorption. This property resembles that of soft tissue. The water content of hydrogels may be as low as a few percent or as high as 99%. Hydrogels are commonly used in medicine to repair skin wounds. Their high water absorption and water retention properties can ensure moisture supply and gas exchange at the wound surface, thus keeping the wound moist and providing a favorable environment for wound healing. Their high water content can also promote the formation of new epithelial tissue and granulation tissue, as well as avoid secondary damage caused by wound adhesion.

Chitosan is an ideal material for wound repair hydrogels. As mentioned above, chitosan has biocompatibility, biodegradability, and antibacterial properties, and it is safe and nontoxic. In addition, chitosan has good hemostatic properties, which are conducive to wound healing; The adhesive properties of chitosan can bond various tissues together, so as to control bleeding and prevent the loss of gas and liquid in the healed tissues. Lisa Myrseth Hemmingsen et al. investigated the properties of chitosan hydrogels for the prevention and control of infection in acute skin injury by combining chitosan with the membrane-targeted antibacterial agent chlorhexidine to enhance the antibacterial activity of the hydrogels. Compared with the formulation without chitosan, the antibacterial effect of chitosan hydrogel on *S. aureus* and *S. epidermidis* was enhanced, indicating that the addition of chitosan synergistically enhanced the antibacterial effect of chlorhexidine. At the same time, chitosan did not show any toxicity toward fibroblasts and keratinocytes [83]. As a hydrogel material, chitosan also has some limitations, such as poor mechanical strength, long gelling time, and poor water solubility. However, the disadvantages can be solved by using chitosan derivatives or adding other substances. Yanjie Wang et al. prepared a novel chitosan hydrogel incorporating graphene oxide [84]. Graphene oxide could improve the mechanical properties of the chitosan hydrogel. Compared with a material without adding the graphene oxide, the tensile stress and strain of the graphene oxide–chitosan hydrogel were increased by 2.1 times and 1.2 times, respectively, and the grafting of the graphene oxide was completed through an amidation reaction. The results of a rat skin defect test showed that the wound healing rate reached 92.2% within 21 days. The composite chitosan hydrogel can provide a reference for other hydrogel studies to improve mechanical properties. As for the potential mechanism of chitosan hydrogels for promoting wound healing, studies have suggested that it may be related to the promotion of chitosan for the synthesis of TP and HYP proteins in granulation tissues, as well as the promotion of collagen fibrillation [85].

### 3.8. Liposomes

Liposomes are spherical vesicles formed by bilayer phospholipid molecules with a diameter of 10–5000 nm. As a carrier for transdermal drug delivery, liposomes have the following advantages: high drug loading, reduced irritation of drugs on the skin, small size and large surface area in contact with the stratum corneum, formation of a film on the skin surface of skin, an encapsulation effect, reduction in water loss, promotion of transdermal permeation of the drug, sustained release, etc.. Chitosan and its derivatives can be used as the coating of transdermal liposomes to increase their stability. Some chitosan derivatives such as *N*-succinyl chitosan can be used as the controlled release shell due to its pH response characteristics, releasing at skin pH [86]. The use of a chitosan coating by Eun-Hye Lee et al. increased the stability of indocyanine green [87]. The stability of the formulation was evaluated by determining the change over time of indocyanine green A780 and FI at room temperature and 37 °C. Free ICG exhibited only 60% and 30% of the initial A780 at room temperature and 37 °C, respectively, and degraded rapidly. In contrast, liposomes significantly delayed the FI loss of ICG. Nevertheless, incorporation of ICG into liposomes failed to completely stabilize ICG, showing that only 10% of the initial FI was retained after 6 days. In contrast, chitosan-coated ICG liposomes showed no significant degradation changes over a test period of at least 6 days. The authors suggested that the stabilization mechanism of chitosan might be related to the electrostatic interaction. In addition, the multilayer structure of chitosan can used to reliably increase the stability of liposomes [88]. Lingli et al. prepared a chitosan-coated liposome for transdermal delivery of lidocaine [89]. The results of the in vitro transdermal permeation test showed that the chitosan-coated lidocaine liposome had a higher permeability coefficient (chitosan-coated lidocaine liposome 11.27 × 10^−3^ cm/h, chitosan-free lidocaine liposome 5.58 × 10^−3^ cm/h, and lidocaine solution 2.12 × 10^−3^ cm/h), as well as more skin deposition, than the chitosan-coated lidocaine liposome and lidocaine solution. This fulfills the clinical need for rapid and local action of lidocaine. Chitosan can increase the adhesion of lidocaine liposomes, thus prolonging the action time of liposomes and the skin, promoting penetration, and improving bioavailability. An in vivo analgesic experiment was also performed to verify the actual effect of the chitosan liposome. The analgesic effect of the drugs was judged by the degree of mouse response to pain, i.e., the time and frequency of licking. The results showed that the licking time and frequency of mice treated with chitosan-coated lidocaine were reduced, showing significant differences compared with the lidocaine liposome and lidocaine solution, thus verifying the penetration-promoting and intradermal retention effects of chitosan on lidocaine liposomes.

### 3.9. Nanometer Bracket

Nano-scaffolds are scaffold-like structures composed of nanofibers. Nanofibers are linear materials with a certain length-to-diameter ratio, with a diameter on the nanometer scale (1–100 nm) and a long length. Nano-scaffolds have a surface effect, as nanofibers with a small particle size have a large surface area, and the surface particles lack the coordination of adjacent atoms; thus, the surface energy is high, and the reaction activity is strong. In addition, the high porosity and low density of nano-scaffolds are favorable for loading a large amount of drugs. Chitosan and its derivatives can modify nano-scaffolds to give them unique properties. Shekh et al. functionalized polyacrylonitrile nanofibers with oxidized chitosan and grafted the antiviral drug acyclovir onto the nanofibers [90]. The modification of oxidized chitosan was verified by scanning electron microscopy; the average diameter increased from 218 to 354 nm, and the surface roughness increased. The in vitro release profiles showed that the acyclovir nanofibers modified by oxidized chitosan exhibited a more sustained release curve than the acyclovir nanofibers without oxidized chitosan modification and the free acyclovir. This phenomenon could be explained by the fact that the oxidized chitosan combined with the drug molecules on the nanofibers to form Schiff bases, which played an anchoring role. For the sustained release of drugs such as acyclovir, reducing its instantaneous high local concentration is very important to control the drug toxicity, and the oxidized chitosan-modified nanofibers can achieve this goal. It should be noted that, in this study, no in vitro skin permeation and in vivo experiments were performed. The actual transdermal effect and therapeutic effect of the oxidized chitosan–acyclovir nanofiber need to be further verified.

## 4. Status and Prospects

Percutaneous administration can avoid the first-pass effect, with long dosing interval and stable blood concentration. It can achieve local or systemic administration, as well as reduce drug stimulation in the gastrointestinal tract. However, many factors limit the clinical application of transdermal drug delivery, including low transdermal efficiency of drugs and intolerable skin irritation. The natural barrier effect of the skin is the greatest clinical challenge faced by transdermal drug delivery. In order to enhance the transdermal penetration of drugs, many different methods and strategies have been developed. Chitosan and its derivatives have been widely used as excipients in transdermal drug delivery systems due to their good biocompatibility, biodegradability, low toxicity, and antibacterial and anti-inflammatory activities. With increasing research, various transdermal drug delivery systems based on chitosan and its derivatives have been continuously developed. Some representative chitosan transdermal formulation research patents are listed in Table 1.

## 5. Conclusions

Chitosan is the only natural cationic polymer with good biocompatibility and biodegradability. Chitosan can be modified by various reactions to obtain a variety of chitosan derivatives with different structures, properties, and functions. Both chitosan and its derivatives have been widely developed for transdermal drug delivery and have demonstrated unique advantages. Transdermal agents based on chitosan and its derivatives exhibit significantly enhanced permeability, which may be associated with one or more of the mechanisms via which chitosan and its derivatives alter the protein structure of the stratum corneum, act on tight junctions in the granular layer, act on intercellular lipids in the stratum corneum, and increase the water content of the stratum corneum. Researchers have widely used certain technical means or processes to apply chitosan and its derivatives to different transdermal dosage forms such as nanoparticles, emulsions, transdermal microneedles, nanocapsules, transdermal patches, transdermal membranes, hydrogels, liposomes, and nano-scaffolds to assist in the transdermal penetration of drugs. In this review, the mechanisms of chitosan and its derivatives in enhancing drug transdermal delivery were discussed in detail, and the specific application forms were summarized, providing theoretical support for the subsequent development of chitosan in transdermal drug delivery systems. Looking forward, with the increasing requirements on the biocompatibility and biosafety of excipients, the research on chitosan and its derivatives will continue. The transdermal preparations based on chitosan can range from small-scale laboratory development to large-scale clinical application.

## Figures and Tables

**Table 1:** Research patents of some representative chitosan transdermal preparations.

Formula	Active Ingredient	Patent Approval Progress	Evaluate	Patent Application Number
Nanoparticle	Insulin	Under trial	The administration system is stable	11/908,599
	Albumin	Lose effectiveness	It is suitable for transdermal administration of macromolecular protein drugs, and it is nontoxic, stable, and controllable	201310289929.6
Emulsion	None	Valid patent	Good emulsification performance and long-term stability	201810245672.7
	Aspirin	Valid patent	Good drug dispersibility	201610598660.3
Transdermal microneedle	Chitosan	Under trial	The microneedle has an antibacterial effect, with a remarkable effect of growth inhibition of *Escherichia coli* and *Staphylococcus aureus*, and it can prevent infection possibly caused in the use proces of the microneedle	201911381855.2
	Stem-cell exosome	Under trial	Solves the problem of transdermal absorption of exosome repair liquid	202011148113.8
Nanocapsule	Fat-soluble drugs such as paclitaxel	Under trial	It has excellent skin permeability and improved bioavailability as compared to oral administration	201980085253.4
Transdermal patch	One or more of citalopram, lorazepam, alprazolam, and olanzapine	Valid patent	Stable and continuous administration; multiple drugs can be delivered simultaneously	202011191501.4
	Wound healing drug	Valid patent	Good air permeability	202121305386.9
Transdermal membrane	Chitosan	Valid patent	Chitosan is easy to release	202110391721.X
Hydrogel	Hyaluronic acid, chitosan	Under trial	The chitosan forms a glue rapidly after azide reaction, and no photoinitiator is needed	202111060377.2
	Salidroside	Under trial	No irritation and allergy, good absorption, and high safety	202111271252.4
Liposome	Quercetin	Valid patent	The liposome is multilayered, the drug is gradually released, and the skin permeability is high	KR1020150080545
Nano-scaffold	Ethyl orthosilicate, triethyl phosphate and calcium nitrate	Valid patent	The chitosan nano-stent has the mechanical properties of high tensile stress, high Young’s modulus, low elongation at break, and high water absorption; it can promote wound vascularization and shorten wound heal time, and it is a skin substitute with good performance	202010654165.6
	Vascular endothelial growth factor (VEGF), follistatin-like 1 (FST-1), antibacterial agents (superparamagnetic iron oxide nanoparticles)	Under trial	The patch simulates the physical and mechanical properties of skin, the therapeutic biomolecules are uniformly distributed, and no additional adhesive is needed, so the patch can quickly repair skin wounds and is also effective for long-term inflammation	17/055,786

Patent data from database https://www.patenthub.cn/ (accessed on 3 March 2022).

## Data Availability

Data sharing not applicable.
